# Disruption of locus coeruleus-related functional networks in Parkinson’s disease

**DOI:** 10.1038/s41531-023-00532-x

**Published:** 2023-05-30

**Authors:** Junyan Sun, Jinghong Ma, Linlin Gao, Junling Wang, Dongling Zhang, Lili Chen, Jiliang Fang, Tao Feng, Tao Wu

**Affiliations:** 1 Center for Movement Disorders, Department of Neurology, Beijing Tiantan Hospital, Capital Medical University, Beijing, China; 2 Department of Neurology, Xuanwu Hospital of Capital Medical University, Beijing, China; 3 Department of General Medicine, Tianjin Union Medical Center, Tianjin, China; 4 Department of Radiology, Guang’anmen Hospital, China Academy of Chinese Medical Sciences, Beijing, China

**Keywords:** Diseases of the nervous system, Parkinson's disease

## Abstract

Locus coeruleus (LC) is severely affected in Parkinson’s Disease (PD). However, alterations in LC-related resting-state networks (RSNs) in PD remain unclear. We used resting-state functional MRI to investigate the alterations in functional connectivity (FC) of LC-related RSNs and the associations between RSNs changes and clinical features in idiopathic rapid eye movement sleep behavior disorder (iRBD) and PD patients with (PD^RBD+^) and without RBD (PD^RBD−^). There was a similarly disrupted FC pattern of LC-related RSNs in iRBD and PD^RBD+^ patients, whereas LC-related RSNs were less damaged in PD^RBD−^ patients than that in patients with iRBD and PD^RBD+^. The FC of LC-related RSNs correlated with cognition and duration in iRBD, depression in PD^RBD−^, and cognition and severity of RBD in patients with PD^RBD+^. Our findings demonstrate that LC-related RSNs are significantly disrupted in the prodromal stage of α-synucleinopathies and proposed body-first PD (PD^RBD+^), but are less affected in brain-first PD (PD^RBD−^).

## Introduction

Parkinson’s disease (PD) is one of the prevalent α-synucleinopathies^1^. The pathological hallmarks of PD include the progressive loss of dopaminergic neurons in the substantia nigra (SN) and the appearance of intraneuronal α-synuclein inclusions (Lewy bodies)^1,2^. In addition, there is a 21–94% loss of locus coeruleus (LC) neurons in patients with PD^3,4^. Based on Braak staging^5^, Lewy pathology in LC precedes SN, and even appears in the prodromal stage of PD^6^. Growing evidence shows that impaired LC contributes to multiple clinical symptoms of PD, such as depression, cognition, and especially rapid eye movement sleep behavior disorder (RBD)^7,8^.

Recently, a dual-hit hypothesis for the origin of PD pathology (body-first vs. brain-first) was proposed^9–11^. According to this hypothesis, in body-first PD, the initial Lewy pathology originates in the peripheral enterovagal system with subsequent caudorostral propagation to the central nervous system (e.g., LC, SN, and cerebral cortex). In contrast, the initial Lewy pathology of brain-first PD emerges in the central nervous system and propagates gradually to the SN and LC. Patients with body-first PD tend to be RBD-positive (PD^RBD+^), whereas those with brain-first PD are more likely to be RBD-negative (PD^RBD−^)^11^.

Up to 94% of patients with idiopathic RBD (iRBD) have Lewy pathology^12^, and eventually develop synucleinopathies^13,14^, mostly PD. Thus, patients with iRBD are considered an ideal cohort for researching the prodromal stage of PD^15^. Moreover, a similar impairment in LC has been detected by neuromelanin-sensitive MRI in patients with iRBD^16^ and PD^RBD+^^17–19^. In contrast, LC is less affected in patients with PD^RBD−^ than those with PD^RBD+^^17^. Dysregulation of noradrenergic circuits has also been associated with various neurological and neuropsychiatric conditions, such as depression and cognitive impairment^20,21^. Loss of noradrenergic nerve terminals originating in the LC has been detected in multiple brain regions in PD with ^11^C-MeNER PET^18,19^. These findings indicate that LC is already damaged in the prodromal stage of PD and is differently affected in body-first and brain-first PD. However, the alterations of LC-related functional networks in the prodromal stage and different phenotypes of PD remain unclear. Thus, we defined LC-related resting-state networks (RSNs) on a whole-brain scale, including the default mode (DMN), executive control (ECN), salience (SAL), sensorimotor (SMN), and visual networks (VIS). Investigation of LC-related RSNs will help understand the neural mechanisms of PD.

Resting-state functional MRI (rs-fMRI) is a powerful method for investigating the functional connectivity (FC) of neural networks in different disease states^22^. Therefore, this study used rs-fMRI to investigate the altered FC of LC-related RSNs in patients with iRBD, PD^RBD−^ and PD^RBD+^. We hypothesized that LC-related RSNs are functionally damaged in patients with iRBD and further deteriorated in patients with PD^RBD+^ (body-first PD), but are less impaired in patients with PD^RBD−^ (brain-first PD). Our findings will help us understand the role of LC in PD pathogenesis.

## Results

### Demographic and clinical assessments

This study finally included 53 patients with iRBD, 64 with PD^RBD−^, 58 with PD^RBD+^, and 69 healthy controls (HCs). There were no significant differences in age, sex, education, duration, and Hoehn and Yahr (H&Y) stage between the groups. There were significant differences in the scores of the Montreal Cognitive Assessment (MoCA), Movement Disorder Society Unified Parkinson’s Disease Rating Scale (MDS-UPDRS) I, Π, and Ш, total Rapid Eye Movement Sleep Behavior Disorder Questionnaire-Hong Kong (RBDQ-HK), Q6-Q12 items of RBDQ-HK (RBD(Q6-Q12)), 17 items Hamilton Depression Scale (HAMD), and Brief Smell Identification Test (B-SIT) between-groups (*p* < 0.01, Bonferroni correction) (Table 1). The between-group differences in the clinical assessments are shown in Table 1. Moreover, voxel-based morphometry (VBM) and head motion parameters were analyzed. We did not find significant differences in intracranial volume (ICV), gray matter volume (GMV), and mean framewise displacement of the head between-groups (*p* < 0.01, Bonferroni correction, Supplementary Table 1).

**Table 1:** Demographic and clinical information of the participants.

variables	HC (*n* = 69)	iRBD (*n* = 53)	PD^RBD−^ (*n* = 64)	PD^RBD+^ (*n* = 58)	ANOVA	post-hoc test					
						HC *vs* iRBD	HC *vs* PD^RBD-^	HC *vs* PD^RBD+^	iRBD *vs* PD^RBD-^	iRBD *vs* PD^RBD+^	PD^RBD-^ *vs* PD^RBD+^
age(years)^a^	62.92 ± 6.89	65.49 ± 8.43	63.33 ± 7.82	63.14 ± 7.2	0.247	-	-	-	-	-	-
sex(M/F)^b^	32/37	32/21	30/34	32/26	0.38	-	-	-	-	-	-
education(years)^a^	11.29 ± 3.41	11.6 ± 2.94	11.14 ± 3.55	11.07 ± 3.08	0.833	-	-	-	-	-	-
UPDRS-I^a^	-	4.58 ± 3.4	7.19 ± 4.22	10.75 ± 6.19	<0.001**	-	-	-	0.02	<0.001**	<0.001**
UPDRS-Π^a^	-	1.21 ± 1.8	8.64 ± 4.72	11.42 ± 6.33	<0.001**	-	-	-	<0.001**	<0.001**	0.004*
UPDRS-Ш^a^	-	4.23 ± 4.47	24.55 ± 12.5	30.37 ± 13.29	<0.001**	-	-	-	<0.001**	<0.001**	0.023
duration(months)^c^	-	60(3–372)	45.88(1.5–254)	48(2–350.25)	0.017	-	-	-	-	-	-
H&Y(I:Π:Ш)	-	-	28:29:7	16:36:6	0.027	-	-	-	-	-	-
MoCA^a^	26.46 ± 2.23	23.88 ± 3.03	24.05 ± 4.32	22.6 ± 3.43	<0.001**	<0.001**	<0.001**	<0.001**	1	0.287	0.11
RBDQ-HK^a^	9.43 ± 6.29	36.83 ± 14.8	9.13 ± 7.86	37.11 ± 14.66	<0.001**	<0.001**	1	<0.001**	<0.001**	1	<0.001**
RBD(Q6-Q12)^c^	0(0-16)	22(4-58)	0(0-16)	20(8-54)	<0.001**	<0.001**	1	<0.001**	<0.001**	1	<0.001**
HAMD^a^	2.48 ± 2.94	5.38 ± 3.87	5.14 ± 3.87	7.8 ± 5.14	<0.001**	<0.001**	0.001*	<0.001**	1	0.012	0.002*
B-SIT^c^	10(8-12)	8(2–11)	8(0–12)	7(0–11)	<0.001**	0.001*	0.001*	<0.001**	1	0.756	1

^a^ANOVA, analysis of variance, mean ± standard deviation, ^b^Pearson χ^2^ test; ^c^Kruskale-Wallis *H* test, median (range).

*HC* healthy control, *iRBD* idiopathic rapid eye movement sleep behavior disorder, *PD*^*RBD−*^ Parkinson’s Disease without RBD, *PD*^*RBD+*^ Parkinson’s Disease with RBD; *M/F*: male/female, *UPDRS* United Parkinson’s disease rating scale, *H&Y* Hoehn & Yahr, I:Π:Ш means 1 to 1.5: 2 to 2.5: 3, *MoCA* Montreal Cognitive Assessment-Beijing Version, *RBDQ-HK* Rapid Eye Movement Sleep Behavior Disorder Questionnaire-Hong Kong, *HAMD* Hamilton Depression Scale, *B-SIT* Brief Smell Identification Test.

**p < 0.001, *p < 0.01, Bonferroni correction.

### Functional connectivity

#### LC-related RSNs

**Fig. 1:**
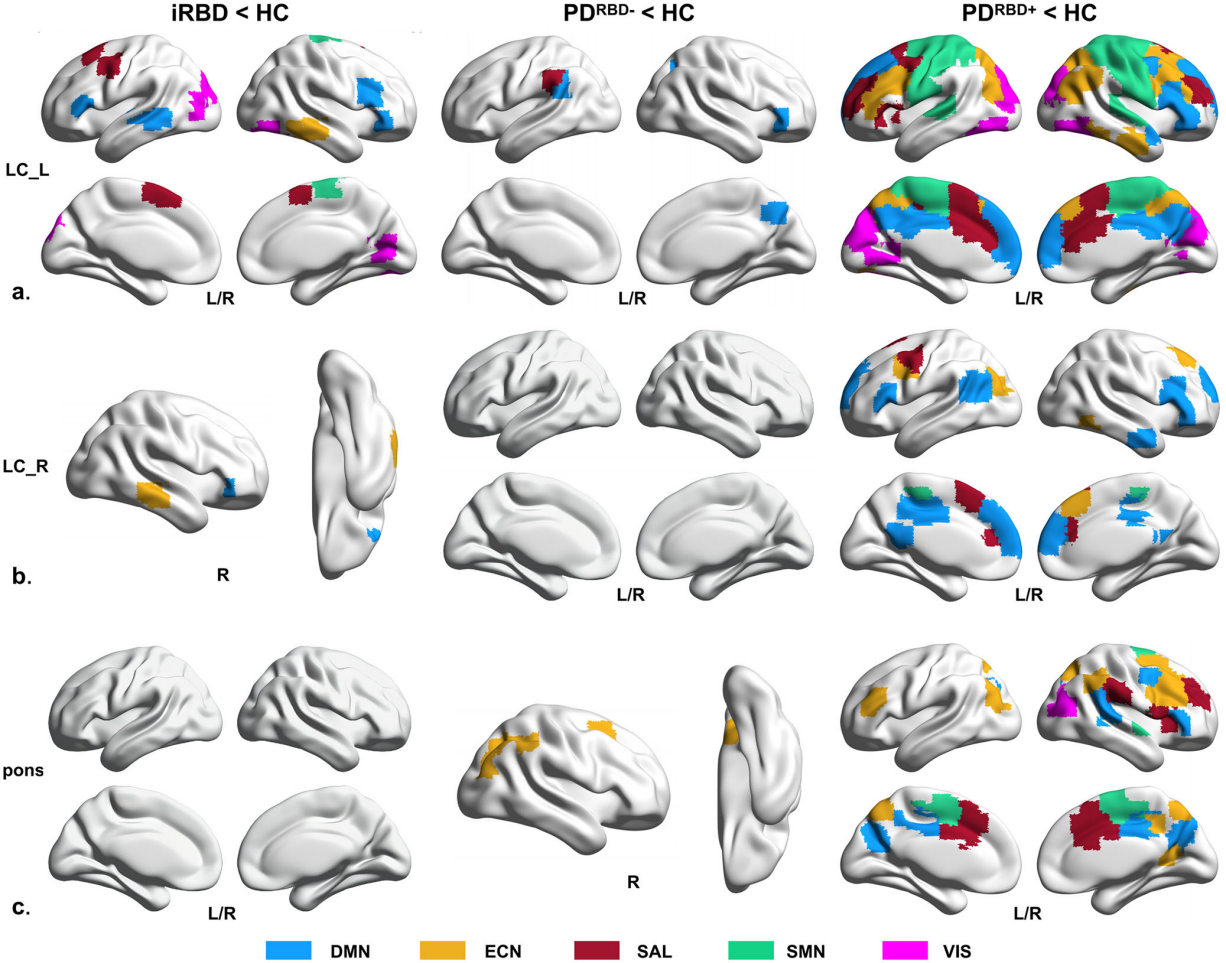
Reduced FC of LC-related RSNs in iRBD and PD patients. **a** Reduced FC patterns of left LC-related RSNs in patients with iRBD and PD. **b** Reduced FC patterns of right LC-related RSNs in patients with iRBD and PD. **c** Reduced FC pattern between pons and RSNs in patients with PD. FC functional connectivity, RSNs rest state networks, HC healthy control, iRBD idiopathic rapid eye movement sleep behavior disorder, PD^RBD−^ Parkinson’s Disease without RBD, PD^RBD+^ Parkinson’s Disease with RBD, LC locus coeruleus, L/R left/right, DMN default mode network, ECN executive control network, SAL salience network, SMN sensorimotor network, VIS visual network, ‘<’: the lower value of functional connectivity than other groups.

We found that patients with iRBD, PD^RBD−^, and PD^RBD+^ had decreased FC of LC-related RSNs compared to HCs (Fig. 1a and Fig. 1b). Specifically, in patients with iRBD, the left LC had decreased FC with all five RSNs, including the DMN, ECN, SAL, SMN, and VIS, whereas the right LC had reduced FC with the DMN and ECN compared to the HCs. Patients with PD^RBD−^ had significantly reduced FC between the left LC and DMN, and SAL compared to the HCs, but had no altered FC between the right LC and any RSNs. Patients with PD^RBD+^ had reduced FC between the left LC and all five RSNs, whereas the right LC had reduced FC with the DMN, ECN, SAL, and SMN compared to the HCs. The detailed results for the brain regions are shown in Supplementary Table 2.

**Fig. 2:**
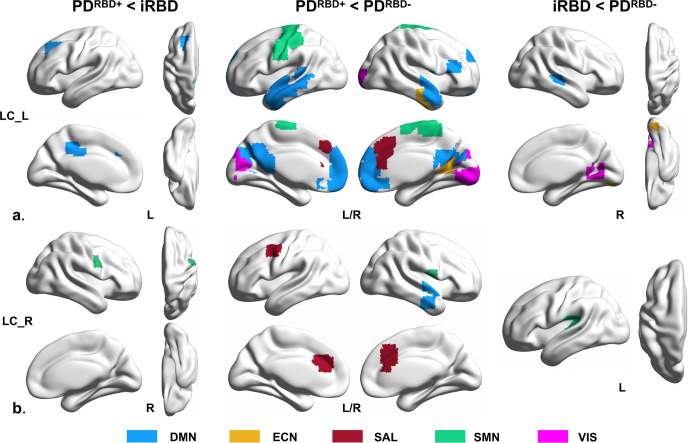
Differences of FC patterns between patient groups. **a** Different FC patterns of left LC-related RSNs in patients with iRBD and PD; **b** Different FC patterns of right LC-related RSNs in patients with iRBD and PD.

The results of the comparisons among the iRBD, PD^RBD−^, and PD^RBD+^ groups are shown in Fig. 2. Compared to patients with iRBD, those with PD^RBD+^ had reduced FC between the left LC and DMN, and between the right LC and SMN. Compared to patients with PD^RBD−^, those with PD^RBD+^ had reduced FC between the left LC and all five RSNs and reduced FC between the right LC and DMN, SAL, and SMN. Interestingly, patients with iRBD had reduced FC between the left LC and DMN, ECN, and VIS, and reduced FC between the right LC and SMN compared to patients with PD^RBD-^. The detailed results for the brain regions are shown in Supplementary Table 3.

#### FC between pons and RSNs

We did not find any differences in FC between patients with iRBD and HCs. Patients with PD^RBD−^ had reduced FC between the pons and the ECN compared to the HCs. Compared to HCs, patients with PD^RBD+^ showed reduced FC between the pons and all five RSNs, similar to the altered FC patterns of LC-related RSNs (Fig. 1c). However, several brain regions had reduced FC with the LC, but not with the pons, such as the angular gyrus, lingual gyrus, paracentral lobule, and superior parietal gyrus. The detailed results of the brain regions are shown in Supplementary Table 4.

#### FC comparison between LC-related RSNs and pons-RSNs

The reduced FC of LC-related RSNs after controlling FC of pons-RSNs are shown in Supplementary Fig. 1. The reduced FC patterns are same before and after controlling the FC of pons-RSNs.

### Correlation analysis

**Fig. 3:**
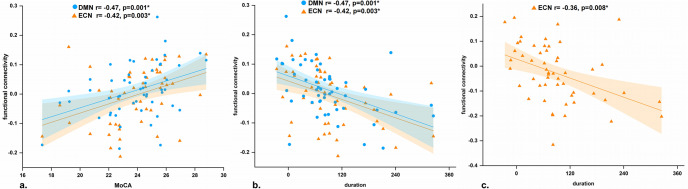
Results of partial correlation analysis in iRBD patients. **a** Functional connectivity of the left LC-DMN/ECN positively correlated with MoCA scores and **b** negatively correlated with disease duration; **c** functional connectivity of the left LC-ECN negatively correlated with disease duration. Bonferroni corrected at *p* < 0.01.

In patients with iRBD, the FC between the left LC and DMN (*r* = 0.4, *p* = 0.006) and ECN (*r* = 0.38, *p* = 0.008) was positively correlated with the MoCA scores (Fig. 3a); in addition, the left LC and DMN (*r* = –0.47, *p* = 0.001) and ECN (*r* = –0.42, *p* = 0.003) were negatively correlated with the disease duration (Fig. 3b), and the FC between the right LC and ECN was negatively correlated with disease duration (*r* = –0.36, *p* = 0.008) (Fig. 3c).

**Fig. 4:**
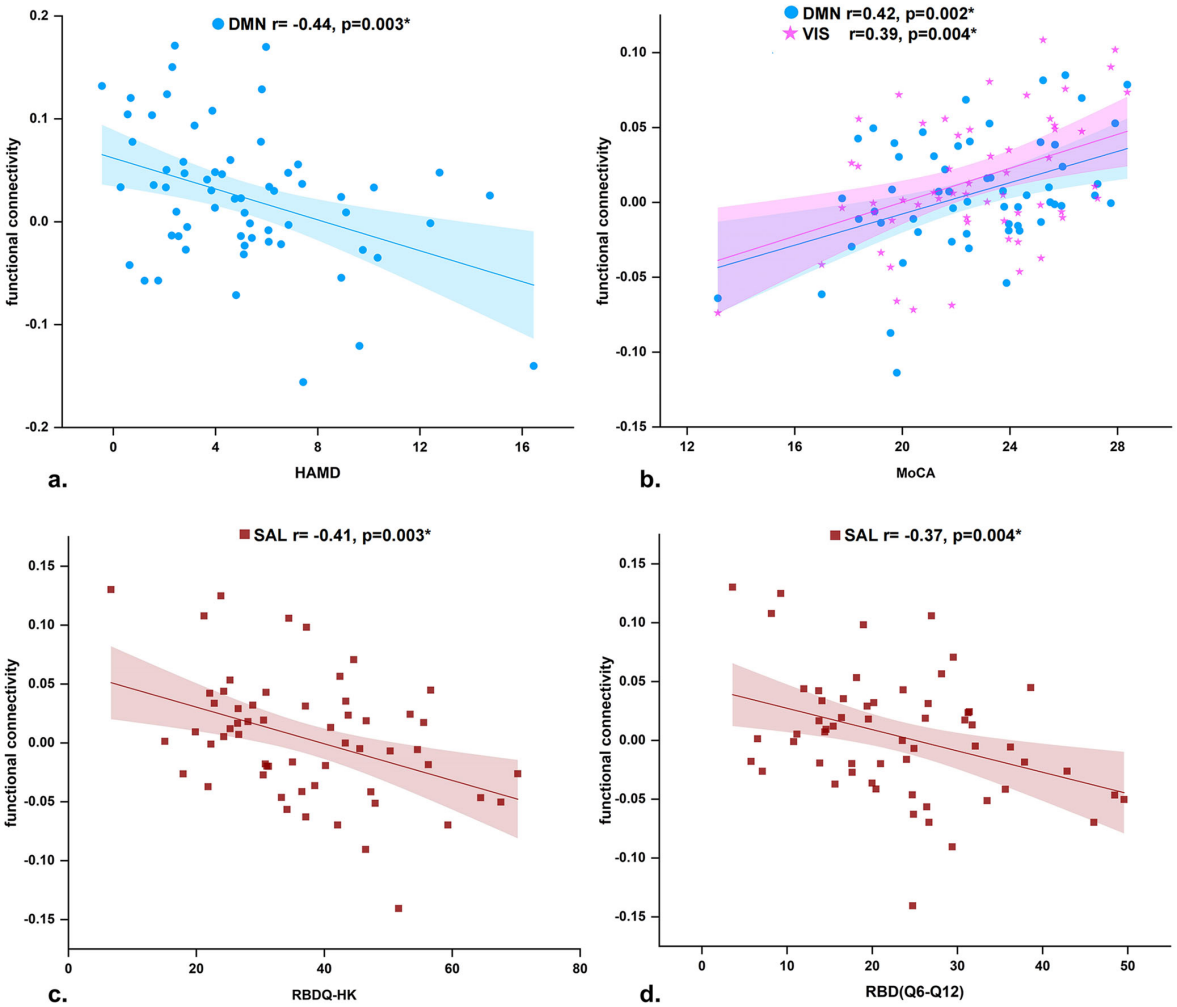
Results of partial correlation analysis in PD patients. **a** Functional connectivity of the left LC-DMN negatively correlated with HAMD in PD^RBD-^ patients; **b** functional connectivity of the left LC-DMN/VIS positively correlated with MoCA scores; **c** functional connectivity of the right LC-SAL negatively correlated with RBDQ-HK, and **d** RBD(Q6-Q12) in PD^RBD+^ patients. Bonferroni corrected at *p* < 0.01.

In patients with PD^RBD−^, FC between the left LC and DMN was negatively correlated with the HAMD scores (*r* = –0.44, *p* = 0.003) (Fig. 4a). In patients with PD^RBD+^, the FC between the left LC and DMN (*r* = 0.42, *p* = 0.002) and VIS (*r* = 0.39, *p* = 0.004) was positively correlated with the MoCA scores (Fig. 4b), whereas the FC between the right LC and SAL was negatively correlated with the total RBDQ-HK scores (*r* = –0.41, *p* = 0.003) (Fig. 4c) and RBD (Q6-Q12) scores (*r* = –0.37, *p* = 0.004) (Fig. 4d).

We did not find significant correlations between clinical assessments and FC between the pons and the five RSNs in patients with iRBD, PD^RBD+^, and PD^RBD−^.

## Discussion

This study is to explore the alterations in LC-related functional networks in the prodromal stage and different phenotypes of PD. As expected, we found a similar disrupted pattern of LC-related RSNs in patients with iRBD and PD^RBD+^, and the disruption in patients with PD^RBD+^ was more severe. However, alterations in FC of LC-related RSNs in patients with PD^RBD−^ were less significant than those in patients with iRBD and PD^RBD+^. Moreover, the FC between the LC and RSNs is relevant to the cognitive impairment and disease duration in patients with iRBD and PD^RBD+^, RBD severity in patients with PD^RBD+^, and depression in patients with PD^RBD−^. Our findings demonstrate that LC-related RSNs are disrupted in the prodromal stage of α-synucleinopathies and are differentially affected in body-first and brain-first PD.

The LC is a small nucleus, and partial volume effects cannot be avoided with the spatial resolution of fMRI used in the current study. We carefully co-registered fMRI images with the LC atlas to control the signal contributions outside the LC. We used Dice similarity coefficients (DSCs) to assess segmentation performance. The results of DSCs were very high across all our participants (mean ± SD: 0.96 ± 0.01, range: 0.96–0.99), showing that the FC variability of LC-related RSNs observed in this study can be largely explained by the intrinsic variations of LC. In addition, we chose the pons as a reference region. We found that the pattern of FC reduction was different between LC-RSNs and pons-RSNs (Fig. 1), and controlling the FC of pons-RSNs did not significantly change the reduced FC patterns of LC-related RSNs (Fig. 1 and Supplementary Fig. 1). Thus, fMRI signals from surrounding tissues likely did not significantly influence our findings.

LC lesions are responsible for the RBD symptom^8,23,24^. Previous imaging studies^17,18^ have identified neuronal loss in the LC and noradrenergic denervation in patients with iRBD. Compared to the HCs, our patients with iRBD exhibited a reduced FC pattern between the LC and all five RSNs. The DMN and ECN are involved in complex cognitive abilities, such as memory retrieval, attention, and language^25–27^. Reduced noradrenalin levels can impair cognitive flexibility and vigilant attention^28^, whereas high noradrenaline levels can restore DMN integrity^29^. Our patients with iRBD had significantly worse MoCA scores than the HCs (Table 1), and the FC of the left LC-DMN and left LC-ECN was positively correlated with cognitive function. These findings show that the disruption of LC-DMN and LC-ECN is associated with the cognitive impairment of patients with iRBD. Previous imaging studies have reported decreased perfusion, metabolism, and FC of hub regions of the DMN^30–32^ (e.g., medial frontal lobe, precuneus, and cingulate), as well as ECN dysfunction^33^, which are linked to cognitive impairment in patients with iRBD. Furthermore, we found that the FC of LC-DMN and LC-ECN was negatively correlated with disease duration, indicating that these networks’ disruption worsens as the disorder progresses.

SAL plays a key role in regulating the dynamic interaction between inward tasks (DMN) and outward tasks (ECN)^34^. The SMN is involved in action planning and execution^35^, whereas the VIS is involved in visual processing^36^. In patients with iRBD, reduced noradrenergic transporter density^37^, dysfunction of SMN^33,38^ and dynamic FC impairments within SMN^39^ and VIS^39,40^ have been reported. However, we did not find that the FC between LC and SAL, SMN and VIS were associated with clinical symptoms of patients with iRBD. A possible reason is that SAL and VIS modulate the attentional and visuospatial systems^41^. However, MoCA assesses global cognitive ability and is not sensitive to specific cognitive domains^42^, such as visuospatial, executive, and attentional functions. More sensitive and specific cognitive tests are warranted in future studies to explore the association between disconnection of LC-SAL/VIS and manifestations in patients with iRBD. In addition, our patients with iRBD exhibited subtle motor impairments (mean UPDRS Ш: 4.23). Patients with iRBD with abnormal cognition and mild motor impairment have a higher risk of converting to α-synucleinopathies^13^. Therefore, longitudinal studies are warranted to explore the relationship between disrupted LC-SMN connectivity and motor symptoms in patients with iRBD and whether disruption of LC-related RSNs can help predict the conversion to α-synucleinopathies.

Our patients with PD^RBD+^ exhibited a similar but worse disrupted FC pattern of LC-related RSNs compared to patients with iRBD. Previous imaging studies have also shown that patients with iRBD have a similar FC pattern of the basal ganglia network^43^, reduced 11C-MeNER binding in the SMN^37,44^, or hypometabolism in cortical areas^45,46^, similar to patients with PD. As Lewy pathology is supposed to be of body-first origin and severely affects LC in both patients with iRBD and PD^RBD+^^11^, it is reasonable that these two groups have a similar disrupted pattern of LC-related RSNs. Compared to patients with iRBD, those with PD^RBD+^ had more severe motor and cognitive impairments (Table 1) and decreased FC between the left LC and DMN, and between the right LC and SMN. Furthermore, patients with PD^RBD+^ have reduced noradrenergic function in the SMN compared to those with iRBD^37^. Our findings show that the disruption of LC-related RSNs is more pronounced in the clinical stage than that in the prodromal stage of body-first PD and may be associated with the progression of clinical symptoms.

Interestingly, the LC-related RSNs in patients with PD^RBD−^ were less affected than those in patients with iRBD and PD^RBD+^ in this study. However, this is consistent with previous reports that patients with PD^RBD−^ showed less reduced LC neuromelanin signals and noradrenergic transporter density than the HCs and patients with PD^RBD+^^11,17,19^. In patients with brain-first PD (PD^RBD−^), Lewy pathology originates in the amygdala and olfactory bulb, whereas the LC is less affected than in patients with body-first PD(PD^RBD+^)^10,11^. Moreover, our patients with PD^RBD-^ had significantly higher HAMD scores than the HCs, and the FC of the left LC-DMN was associated with depression. Reduced FC of the DMN has been detected in patients with depression^47^ and PD with depression^48^. Additionally, this suggests that dysfunction of the DMN associated with noradrenergic denervation might be one of the reasons for depression in patients with PD^RBD−^.

Although our patients with iRBD and PD showed a loss of the sense of smell, no correlation was observed between B-SIT scores and FC of LC-related RSNs. The LC sends noradrenergic projections to the olfactory bulb and olfaction-related forebrain cortex, such as the piriform, entorhinal, and orbitofrontal cortex^49^, and impaired LC is associated with olfactory dysfunction^50^. However, multiple factors are known to be involved in olfactory dysfunction in PD, such as the deficiency of dopamine and acetylcholine^51,52^. Thus, we will consider combining neural networks related to multiple transmitters (such as dopamine and noradrenaline) in the future to investigate the neural mechanisms underlying olfactory dysfunction in PD.

We found that the disruption of the FC between the left LC-related RSNs was more severe than that between the right LC in all three patient groups. As most of our patients with PD (76/122) exhibited right-side dominant motor symptoms, damage to the left hemisphere was more pronounced. The fiber projections of the LC neurons showed an ipsilateral predominance^53,54^. Our finding is consistent with previous reports of left-hemispheric dominance of nigrostriatal deficits in right-handed iRBD^31,55^. This finding suggests that the neurodegenerative process may begin asymmetrically during the prodromal stage of α-synucleinopathies, initially in the dominant hemisphere. In addition, there were no significant differences in brain volume among the groups, and we used the VBM results as variables of no interest in statistical analysis. Thus, structural changes in patients with iRBD and PD are unlikely to confound our findings.

This study had some limitations that should be acknowledged. First, this is a cross-sectional study. A longitudinal study is necessary to assess whether disruption of LC-related RSNs in iRBD is associated with conversion to PD and whether the progression of LC impairments is different in patients with PD^RBD+^ and PD^RBD−^. Second, the current study does not include some non-motor symptoms (such as autonomic dysfunction and anxiety). Future studies must clarify the association between LC impairments and these manifestations in patients with iRBD and PD. Third, given the small volume of the LC and the spatial resolution of rs-fMRI in this study, partial volume effects cannot be avoided. Using a higher field strength scanner and acquiring higher spatial resolution fMRI data in the future will help eliminate partial volume effects. Finally, neuromelanin-sensitive MRI helps localize the LC^56^, which will be applied in future studies to optimize LC localization.

In conclusion, our work explored the FC patterns of LC-related RSNs in patients with iRBD and PD. LC-related RSNs are significantly disrupted in prodromal and proposed body-first PD (PD^RBD+^), but are less affected in brain-first PD (PD^RBD−^). Dysfunction of LC-related RSNs may contribute to the clinical symptoms in patients with iRBD and PD.

## Methods

### Participants

In this study, 129 patients with idiopathic PD (60 PD^RBD+^ and 69 PD^RBD−^) and 55 patients with iRBD were recruited. Patients with PD were diagnosed according to the MDS Clinical Diagnostic Criteria^2^. Patients with PD were clinically defined as PD^RBD+^ according to the following criteria^57^: (1) patients or their bedmates had clear complaints of abnormal behavior during nocturnal sleep, (2) total score of RBDQ-HK ≥ 19, and (3) score ≥ 8 on RBD (Q6–Q12). Patients with PD who did not meet the above criteria were defined as having PD^RBD−^. Patients with iRBD were screened using international diagnostic criteria^58^. Clinically defined patients with iRBD and PD^RBD+^ were examined using polysomnography (PSG). Only patients who confirmed by PSG were included in the current study. In addition, 72 age- and sex-matched HCs were recruited. The inclusion criteria for the HCs were no evident neuropsychiatric symptoms, structural MRI abnormalities, or subjective memory complaints. All the participants were right-handed. This study was performed in accordance with the Declaration of Helsinki and was approved by the Ethics Committee of Beijing Tiantan Hospital, Capital Medical University. All participants signed informed consents prior to the experiment.

### Clinical assessments

All participants were evaluated using MoCA, 17-items HAMD, and B-SIT scores. Patients were assessed using the MDS-UPDRS and RBDQ-HK. In addition, the H&Y stage was evaluated in patients with PD. PD patients were evaluated while not taking any anti-parkinsonian medication for a minimum of 12 h.

### MRI data acquisition

MRI data were acquired using a 3.0 T scanner (Siemens, skyra, Germany). Preparation of participants before scanning included stabilization of the head with foam pads, reduced audible noise with earplugs, and closed eyes that remained awake during scanning. Three-dimensional T1-weighted images were obtained with a magnetization-prepared rapid acquisition gradient echo (MP-RAGE) sequence: repetition time (TR) = 2530 ms, echo time (TE) = 2.98 ms, flip angle (FA) = 7°, field of view (FOV) = 224 mm × 256 mm, matrix = 256 × 256, voxel size = 1 × 1 × 1 mm^3^, slice thickness = 1 mm, number of slices = 192 (sagittal), no gap, and scanning time = 5 min 13 s. The rs-fMRI data were obtained with gradient echo planar imaging (EPI) sequence: TR = 2000 ms, TE = 30 ms, FA = 90°, FOV = 220 mm × 220 mm, matrix = 64 × 64, voxel size = 3.4 × 3.4 × 3.6 mm^3^, number of slices = 35 (axial), slice thickness = 3.5 mm (no gap), time points = 176, and scanning time = 5 min 52 s.

### MRI data preprocessing

First, the CAT12 (Computational Anatomy toolbox) was used for the analysis of VBM with T1-weighted images (http://dbm.neuro.uni-jena.de/cat12/), which was based on the MATLAB R2018b platform (http://www.mathworks.com). The analysis process included spatial normalization, bias-field correction, tissue segmentation, and modulation. The ICV and GMV of all participants were measured in normalized space.

Preprocessing of rs-fMRI data was performed using the DPARSF6.0 toolbox (Data Processing Assistant for Resting-State fMRI, version 6.0) (http://www.restfmri.net) based on MATLAB 2018b, including removal of the first 10 time points, slice-timing, realign, regressing off cerebrospinal fluid (CSF) and global signals and Friston-24 head motion parameters, and normalization (voxel size 3 × 3 × 3 mm^3^) using the DARTEL (Diffeomorphic Anatomical Registration using Exponentiated Lie algebra) algorithm. To assess the segmentation performance, the DSCs between gold standard (S) and image segmentation results (I) were calculated as: *DSC* = *2 (I* ∩ *S) / (I* + *S)*. *∩* represents the overlap of the gold standard and image segmentation results. DSCs were very high across all participants (mean ± SD: 0.96 ± 0.01, range: 0.96–0.99). Participants with excessive head motion (>2.5 mm of maximal translation in any direction, 2.5° of maximal rotation, or mean framewise displacement > 0.25 mm) were excluded^59^. Finally, we excluded 2, 5, and 2 patients with iRBD, PD^RBD−^, PD^RBD+^, respectively, and 3 HCs.

As the LC is located in the brainstem and adjacent to the fourth ventricle, fMRI signals of the LC may be confounded by physiological noise (cardiac and respiratory cycles). Thus, we used MELODIC (Multivariate Exploratory Linear Optimized Decomposition into Independent Components) (https://fsl.fmrib.ox.ac.uk/fsl/fslwiki/MELODIC), a toolbox included in FSL (FMRIB’s Software Library), to perform ICA analysis on the normalized rs-fMRI images to removing physiological noises^60^. Two raters (J.S and J.W) performed ICA analysis, according to the guidelines of “hand classification of fMRI ICA noise components” to accurately identify the signal components of the LC^61^. Specifically, we first identified ICA decomposition (spatial maps, time series, and power spectra). An innocent signal was defined as follows: first, the spatial map of one component should be featured with clear resting state networks or nodes and away from the main veins, white matter, or CSF; second, the time series should be oscillatory during the time course, without sudden or abrupt changes; and finally, the power spectra should be predominantly distributed at low frequencies (at least one strong peak within 0.01–0.1 Hz) (for example, Supplementary Fig. 2). The components that did not meet the above criteria were removed as noise, such as head motion noise, cardiac and respiratory noise, and CSF pulsation (Supplementary Fig. 3). There was high inter-rater reliability between the two raters. When disagreements appeared, the two raters reached a consensus through discussion. The noise components selected by both raters were removed. The noise from CSF pulsation spatially overlapped with the 3rd and 4th ventricles (Supplementary Fig. 3). A noise-containing components from the 4th ventricle was identified for each participant.

The rs-fMRI images were then low-pass filtered (0.01 ~ 0.1 Hz). Considering the small volume of LC, rs-fMRI images were smoothed with a 3 mm full-width at half maximum (FWHM) Gaussian kernel^62^. Bilateral LCs were defined according to a probabilistic LC atlas (5% probability) based on ultra-high-field 7 T neuromelanin-sensitive MRI data from 53 healthy participants (mean age, 66 years; range, 52–84 years), applicable to neurodegenerative diseases^63^. LC sends noradrenergic projections to extensive brain networks^64^, including the default mode network (DMN)^25,26,65^, executive control network (ECN)^33,66^, salience network (SAL)^34^, sensorimotor network (SMN)^35^, and visual network (VIS)^36^ which have been reported to be damaged in PD^67,68^. As we aimed to investigate whether impaired LC contributes to the damage of brain networks, resulting in related clinical symptoms, these five networks were chosen as LC-related RSNs for FC analysis in the current study. As RSNs derived from older adults have spatiotemporal configurations different from those of young adults^69,70^, we primarily defined the five LC-related RSNs according to the Atlas55 + , which is a brain functional atlas^69^ is generated from late adulthood and covers the whole brain. The DMN mainly includes the medial prefrontal cortex/ventral anterior cingulate cortex, precuneus/posterior cingulate cortex, inferior frontal cortex, and middle temporal cortex; the ECN contains parts of the dorsolateral prefrontal cortex, lateral and medial parietal cortex, posterior inferior temporal cortex, and part of the cerebellum; the SAL comprises the dorsal anterior cingulate cortex, anterior supplementary motor area, supramarginal gyri, part of subcortical regions, and cerebellum; the SMN covers the sensory and motor regions (precentral and postcentral gyrus and supplementary motor area), and thalamus; and the VIS largely covers the occipital lobe^69^. The DMN, ECN, and SAL are involved in higher-order cognitive and psychological functions as part of the intrinsic system, whereas the VIS and SMN support specialized visual, sensory, and motor processing as part of the extrinsic system. FC was measured as a correlation of activity in the bilateral resampled LCs (separately) with all voxels in these RSNs (bilateral). The correlation coefficients were converted to *z* values using Fisher’s *r*-to-*z* transformation.

Given the small volume of LC and the spatial resolution of rs-fMRI, there were potential partial volume effects. Therefore, we chose the pons as a reference region to calculate the FC between the pons and RSNs, and directly compared the FC between LC-RSNs and pons-RSNs ((LC_patients_ − LC_HC_) − (pons_patients_ − pons_HC_)) in order to validate that fMRI signals from surrounding regions did not significantly contaminate the FC results of LC-related RSNs. The pons was defined on the isotropic International Consortium for Brain Mapping (ICBM152) T1-weighted template^71^ using the ITK-SNAP software (http://www.itksnap.org/pmwiki/pmwiki.php).

### Statistical analysis

The IBM-SPSS v25.0 software (Armonk, New York, USA) was used for variable analysis. For neuropsychological assessments, the normality of the variables was confirmed using Q-Q plots and Kolmogorov–Smirnov tests. Continuous variables were analyzed using one-way analysis of variance (ANOVA) and were expressed as mean ± SD (standard deviation). Other variables were analyzed using non-parametric tests and were expressed as medians (ranges). Pearson’s *χ*^*2*^ test was used to analyze sex differences. The Bonferroni correction was used for multiple comparisons (*p* < 0.01).

One-way analysis of covariance (ANCOVA) was used to calculate the differences in FC among the groups, with age, gender, education, mean framewise displacement, ICV, and GMV of participants as covariates of no interest. Post-hoc tests (with the same covariates as those for the ANCOVA analysis) were performed to explore between-group differences in FC. Family-wise error (FWE) correction was used for multiple comparisons (permutation test set to 20,000, cluster *p* < 0.05, with a cluster extent of ≥10 voxels, and voxel *p* < 0.001). Partial correlation analysis (with the same covariates as those for ANCOVA analysis) was used to analyze the relationship between FC and clinical assessments (*p* < 0.01, Bonferroni corrected).

### Reporting summary

Further information on research design is available in the Nature Research Reporting Summary linked to this article.

## Supplementary information

Supplementary file

Reporting Summary

## Data Availability

The dataset used in this study is available for qualified researchers to request by contacting the corresponding author.

## Code availability

CAT12; DPABI (v6.0, http://www.rfmri.org/dpabi); FSL- MELODIC (https://fsl.fmrib.ox.ac.uk/fsl/fslwiki/MELODIC); ITK-SNAP (http://www.itksnap.org/pmwiki/pmwiki.php); MATLAB R2018b (http://www.mathworks.com); SPM12 in the study are open sources.
