# Supplementary material for: Disruption of locus coeruleus-related functional networks in Parkinson’s disease
Source: NPJ Parkinsons Dis. 2023 May 30;9:81. doi: 10.1038/s41531-023-00532-x (PMC10229645; doi:10.1038/s41531-023-00532-x)
Supplement: Supplementary file 1 — Supplementary file [file 41531_2023_532_MOESM1_ESM.pdf]

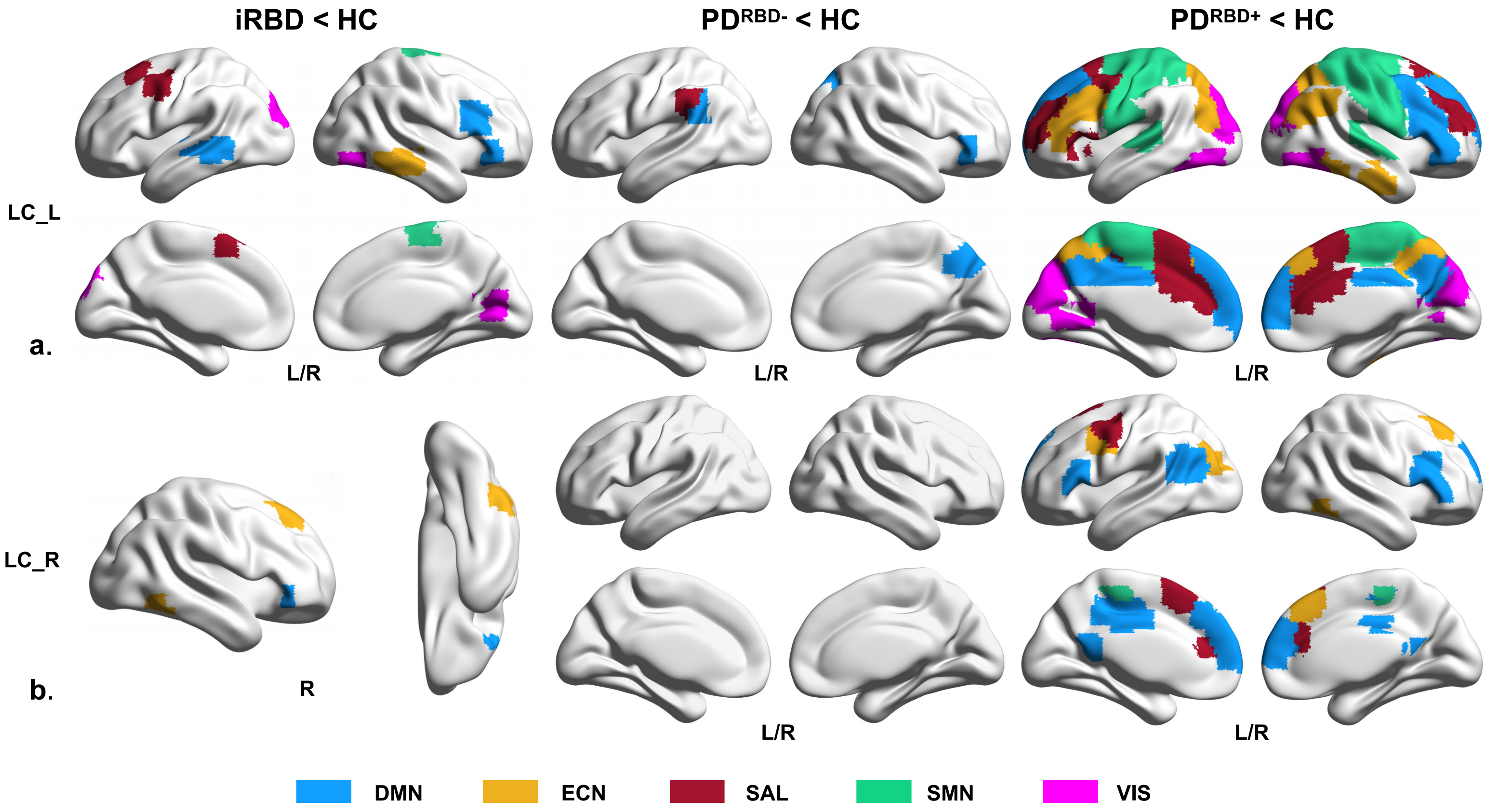

Supplementary Figure 1. Reduced FC of LC-related RSNs in iRBD and PD patients after controlling FC of pons-RSNs. (a) Reduced FC patterns of left LC-related RSNs in patients with iRBD and PD after controlling FC of pons-RSNs; (b) Reduced FC patterns of right LC-related RSNs in patients with iRBD and PD after controlling FC of pons-RSNs.

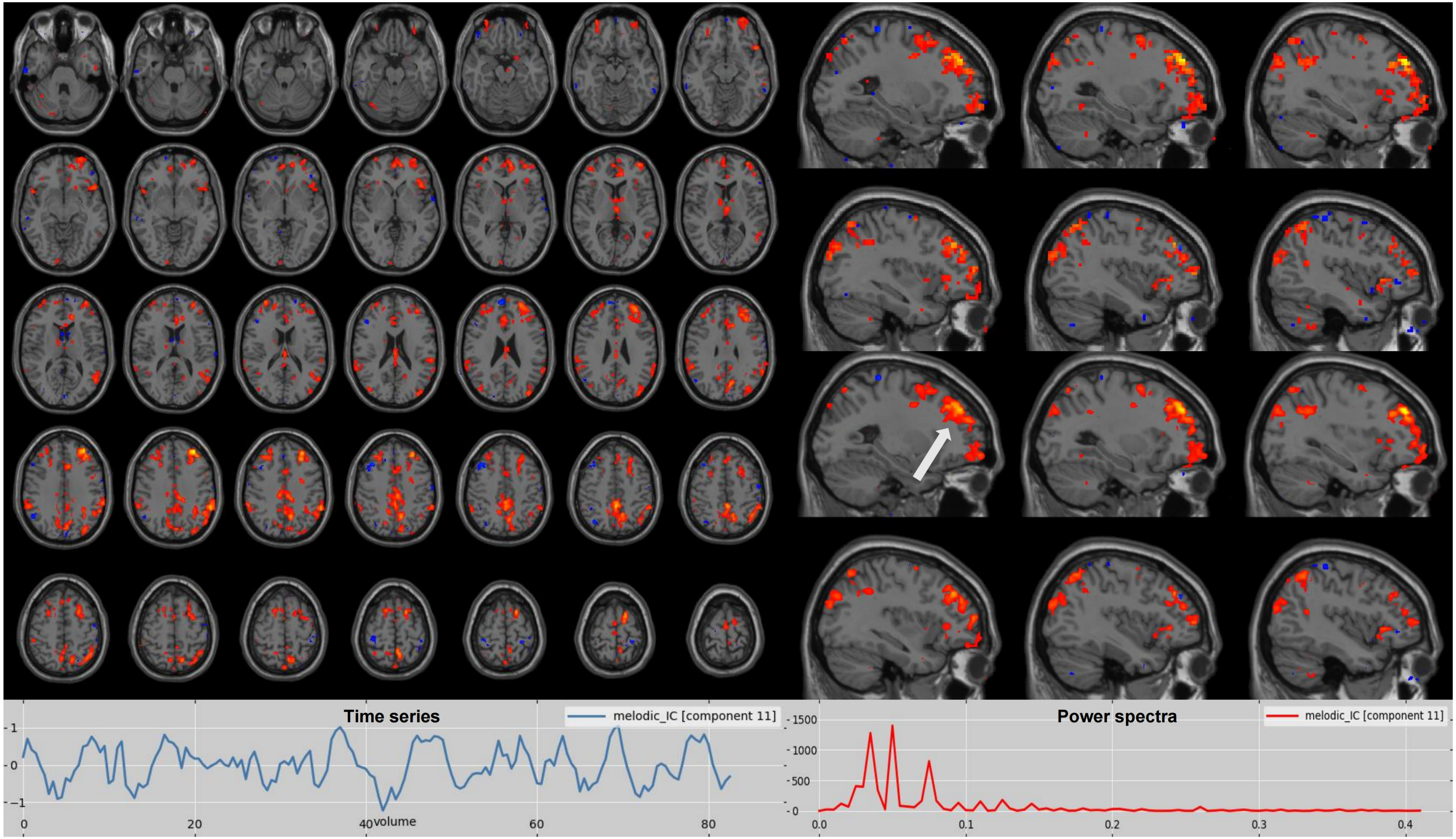

Supplementary Figure 2. Innocent signal of ICA classification. The arrow indicates the innocent signal of frontal lobe.

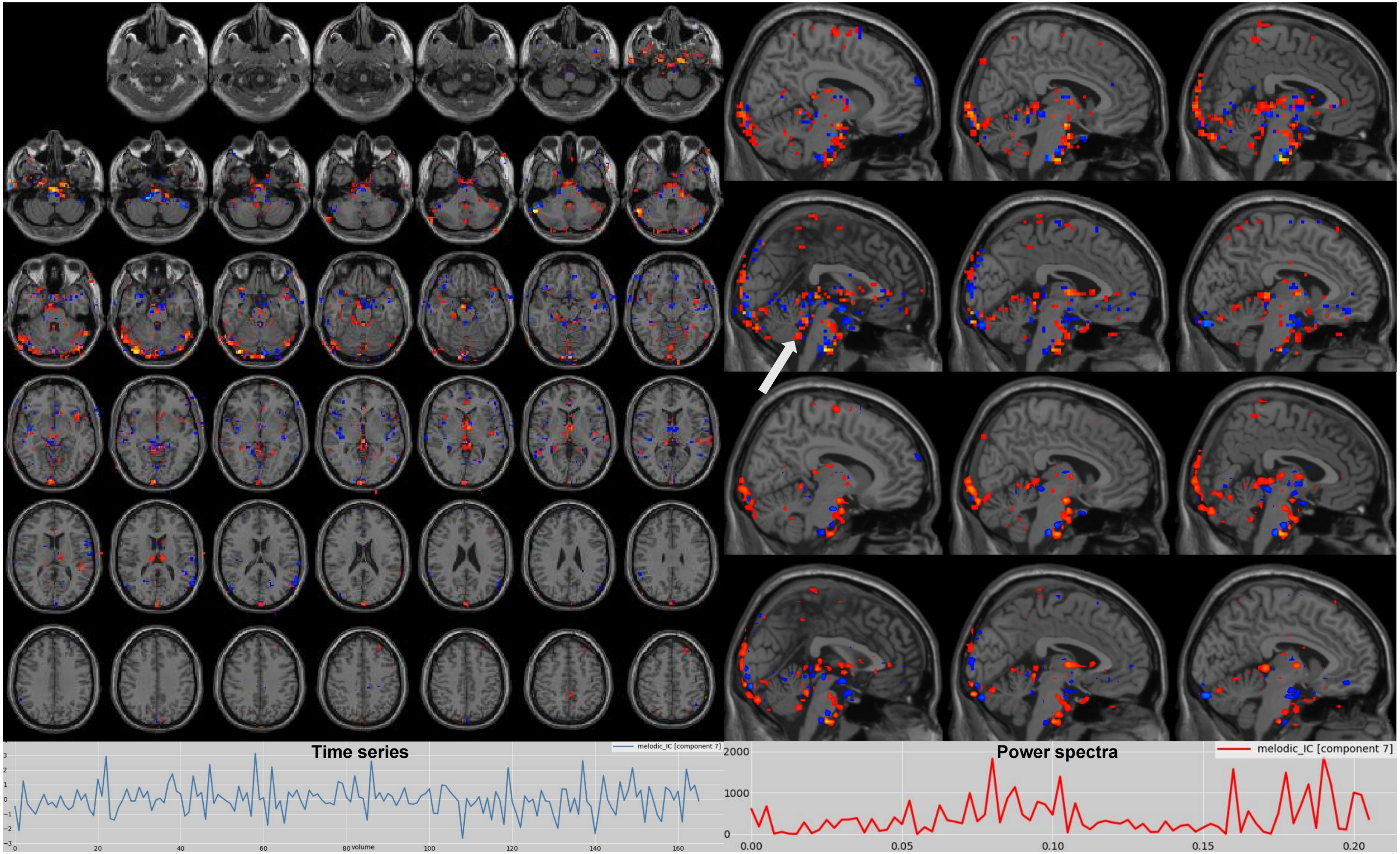

Supplementary Figure 3. The noise component of ICA classification. The arrow indicates the cerebrospinal fluid pulsation noise in the 4th ventricle.

Supplementary Table 1. Results of VBM analysis and head motion parameters.

variables	HC (n=69)	iRBD (n=53)	PD^{RBD-} (n=64)	PD^{RBD+} (n=58)	ANOVA
ICV	1434.63±138.67	1469.62±142.68	1453.06±148.43	1477.42±130.76	0.329
GMV	629.85±56.7	633.1±54.61	618±51.15	619.78±49.22	0.325
mean framewise displacement	0.09±0.05	0.09±0.05	0.09±0.06	0.08±0.05	0.423

VBM: voxel-based morphometry; ANOVA: analysis of variance; ICV: intracranial volume; GMV: grey matter volume.

*p<0.01, Bonferroni correction.

Supplementary Table 2. Decreased functional connectivity of LC-related RSNs in iRBD and PD patients.

seeds	RSNs	cluster size	AAL	L/R	BA	peak intensity (T value)	MNI coordinate X	Y	Z
**iRBD < HC**									
LC_L	DMN	41	inferior frontal gyrus, orbital part	R	47	-4.89	51	27	-9
		10/31	inferior frontal gyrus, triangular gyrus part	L/R		-4.05	51	24	21
		25	middle temporal gyrus	L		-4.85	-48	-24	0
	ECN	13	middle temporal gyrus	R		-4.05	66	-24	-9
		11	inferior temporal gyrus	R		-4.3	51	-42	-18
	SAL	19	cerebellum_crus1	R		-4.63	48	-51	-30
		33	precental gyrus	L		-4.24	-36	0	51
		32	supplementary motor area	L	6	-4.48	-9	15	60
		12	superior frontal gyrus, dorsolateral	L		-4.16	-12	15	57
	SMN	15	supplementary motor area	R		-3.73	12	-21	72
	VIS	23	calcarine fissure and	R		-4.51	3	-66	9
		27	inferior occipital gyrus	R		-4.47	39	-75	-21
		12	middle occipital gyrus	L		-3.97	-36	-78	6
		19	superior occipital gyrus	L		-4.29	-21	-90	21
LC_R	DMN	13	inferior frontal gyrus, orbital part	R		-4.65	51	27	-9
	ECN	15	middle temporal gyrus	R		-3.99	60	-33	-9
**PD^{RBD-} < HC**									
LC_L	DMN	15	inferior frontal gyrus, orbital part	R		-3.74	51	30	-6
		20	precuneus	R		-4.26	12	-63	45
		15	supramarginal gyrus	L		-4.01	-57	-45	24
	SAL	20	supramarginal gyrus	L		-4.32	-60	-39	33
**PD^{RBD+} < HC**									
	DMN	23	anterior cingulate and paracingulate gyri	R		-5.18	3	45	18
		153/22	median cingulate and paracingulate gyri	L/R	31/24	-6.15	-6	-36	39
		19	inferior frontal gyrus, opercular part	R		-4.67	51	18	12
		74	inferior frontal gyrus, orbital part	R	47	-5.19	51	30	-9
		130	inferior frontal gyrus, triangular gyrus part	R	47/46	-5.23	54	27	18
		55/55	middle frontal gyrus	L/R	8	-4.77	-36	24	36
		219/125	superior frontal gyrus, dorsolateral_medial	L/R	9/10	-6.2	3	57	9
		101/61	superior frontal gyrus, dorsolateral	L/R	8	-5.31	18	51	30
		18	precental gyrus	R		-4.73	45	3	39
		45/89	precuneus	L/R	7/31	-5.47	3	-42	45
		27	temporal pole: superior temporal gyrus	R	38	-4.7	57	9	-9
	ECN	44	angular gyrus	R		-4.54	27	-60	48
		16	cerebellum_crus1	L		-4.23	-30	-75	-21
		18	median cingulate and paracingulate gyri	L		-6.14	-12	-39	42
		59	inferior frontal gyrus, triangular gyrus part	L		-4.6	-36	27	27
		39	inferior frontal gyrus, opercular part	R		-4.84	51	15	33
		52/98	middle frontal gyrus	L/R	9	-4.72	-45	12	36
		23/39	superior frontal gyrus, dorsolateral_medial	L/R	8	-5.33	-6	24	54
		17	superior frontal gyrus, dorsolateral	R		-3.98	27	-3	51
		123/66	middle occipital gyrus	L	19/39	-4.96	-27	-84	30
		19	superior occipital gyrus	R		-4.57	30	-78	39
		32/29	superior parietal gyrus	L/R	7	-4.8	-15	-69	54

		48/22	precental gyrus	L/R	9	-5.99	-48	6	42
		61/89	precuneus	L/R	7	-5.48	6	-48	45
		67	inferior temporal gyrus	R	37/20	-5.35	57	-57	-18
LC_L		23	cerebellum_crus1	R		-4.34	48	-54	-27
		66/39	anterior cingulate and paracingulate gyri	L/R	32	-5.19	-6	30	27
		48/22	median cingulate and paracingulate gyri	L/R		-5.36	-3	-27	45
		114/87	middle frontal gyrus	L/R	9	-5.47	27	39	21
	SAL	36	superior frontal gyrus, dorsolateral_medial	L	9	-4.71	-3	42	27
		58/35	superior frontal gyrus, dorsolateral	L/R	10	-5.47	27	39	21
		92	precental gyrus	L	6	-5.71	-36	0	51
		103/57	supplementary motor area	L/R	6/8	-6.04	-3	21	54
		30/14	median cingulate and paracingulate gyri	L/R	31	-5.39	-12	-42	51
		38	superior frontal gyrus, dorsolateral	R	6	-5.54	12	-12	75
		116/76	paracentralobule	L/R	6/5	-5.16	-3	-30	54
		18	inferior parietal, but supramarginal gyrus and angular gyrus gyri	L		-4.37	-27	-42	51
		19/29	superior parietal gyrus	L/R	7	-4.9	18	-45	66
	SMN	266/227	postcentral gyrus	L/R	/4/6/4	-5.85	48	-12	36
		97/195	precental gyrus	L/R	6	-4.42	-27	-6	57
		37/38	precuneus	L/R	7	-5.5	-12	-45	54
		41	rolandic operculum	R	6	-5.07	54	-3	15
		65/98	supplementary motor area	L/R	6	-5.87	9	-9	75
		29/36	superior temporal gyrus	L/R		-4.76	-60	-30	9
		20	calcarine fissure and surrounding cortex	L		-4.9	3	-66	9
		31/33	cuneus	L/R		-4.64	0	-81	36
		17	lingual gyrus	L		-4.53	0	-66	6
	VIS	‘12/13	inferior occipital gyrus	L/R	19	-4.51	-51	-75	-12
		52/36	middle occipital gyrus	L/R	19	-5.07	-24	-84	24
		80/71	superior occipital gyrus	L/R	7/19	-5.63	-24	-84	27
		32	angular gyrus	L		-4.95	-48	-66	24
		21	anterior cingulate and paracingulate gyri	R		-6	6	45	21
		67	median cingulate and paracingulate gyri	L	31	-4.58	-3	-36	45
	DMN	37	inferior frontal gyrus, orbital part	R	47	-5.27	51	36	-15
		16/39	inferior frontal gyrus, triangular gyrus part	L/R		-4.65	54	27	18
		30	superior frontal gyrus, dorsolateral	L		-4.66	-18	45	30
		85/66	superior frontal gyrus, dorsolateral_medial	L/R	9/10	-5.88	3	45	21
LC_R		53	middle temporal gyrus	L	39	-5.01	-51	-54	21
		35	middle occipital gyrus	L		-4.26	-36	-72	30
	ECN	20	precental gyrus	L		-4.63	-48	6	42
		18	inferior temporal gyrus	R		-5.25	57	-57	-21
		19	anterior cingulate and paracingulate gyri	R		-5.52	3	42	21
	SAL	37	precental gyrus	L	6	-4.77	-48	6	45
		22	supplementary motor area	L		-5.07	-6	21	54
	SMN	17	paracentral lobule	L		-4.32	-6	-24	48

RSNs： resting-state networks; AAL: Anatomical Automatic Labeling; L/R: left/right; BA: Brodmann area; MNI: Montreal Neurological Institute; LC_L/R: left and right locus coeruleus; DMN: default mode network; ECN: executive control network; SAL: salience network; SMN: sensorimotor network; VIS: visual network; ‘<’: the lower value of functional connectivity than other groups.

Supplementary Table 3. Altered functional connectivity of LC-related RSNs between patient groups.

seeds	RSNs	cluster size	AAL	L/R	BA	peak intensity (T value)	MNI coordinate		
							X	Y	Z
			**PD^{RBD+} < iRBD**						
LC_L	DMN	19	median cingulate and paracingulate gyri	L		-4.47	-6	-36	39
		12	superior frontal gyrus,dorsolateral	L		-4.5	-18	33	36
LC_R	SMN	12	precentral gyrus	R		-4.7	60	3	33
			**PD^{RBD+} < PD^{RBD-}**						
LC_L	DMN	17	anterior cingulate and paracingulate gyri	R		-4.44	6	48	18
		10	calcarine fissure and surrounding cortex	L		-4.25	-6	-45	6
		16	cerebelum_crus2	L		-4.72	-18	-81	-33
		21	posterior cingulate gyrus	L		-4.3	-6	-51	21
		49	cuneus	L	31/7	-4.96	0	-72	27
		16	inferior frontal gyrus, triangular gyrus part	R		-4.46	54	33	15
		52/11	superior frontal gyrus, medial orbital	L/R	11/10	-5.29	-9	60	-9
		56/32	superior frontal gyrus, orsolateral_medial	L/R	9/10/32	-5.18	3	48	6
		68/28	precuneus	L/R	31	-4.52	-3	-48	12
		20	rectus	R	11	-4.52	6	48	-18
		21	inferior temporal gyrus	L		-4.63	-48	-6	-39
		88/34	middle temporal gyrus	L/R	21	-5.47	54	-9	-24
		10/32	superior temporal gyrus	L/R	21	-5.8	63	-3	-3
	ECN	23	calcarine fissure and surrounding cortex	R		-4.41	15	-54	12
		14	cerebellum_crus1	R		-4.05	9	-81	-27
		10	cerebellum_crus2	R		-4.29	9	-81	-30
		20	inferior temporal gyrus	R		-5.08	54	-9	-27
	SAL	11	anterior cingulate and paracingulate gyri	R		-3.73	3	42	18
		24	median cingulate and paracingulate gyri	R		-4.14	12	27	30
	SMN	23/43	paracentralobule	L/R	6	-5.64	9	-30	72
		83	postcentral gyrus	L	3	-4.76	-33	-30	48
		35/10	precental gyrus	L/R	4	-5.77	12	-33	72
		26	supplementary motor area	R	6	-4.43	9	-12	75
		18	superior temporal gyrus	R		-4.94	60	-6	0
	VIS	46	lingual gyrus	R		-4.38	12	-99	-9
		23/30	calcarine fissure and surrounding cortex	L/R	17	-4.42	3	-90	-9
		20	cuneus	L		-5.66	0	-81	36
LC_R	DMN	15	inferior temporal gyrus	R		-5.15	60	-9	-27
		24	superior temporal gyrus	R	21	-5.18	63	-3	-3
	SAL	18	anterior cingulate and paracingulate gyri	L	32	-4.31	-9	27	27
		31	median cingulate and paracingulate gyri	R		-4.4	9	24	30
		19	precental gyrus	L		-5.23	-45	6	48
	SMN	25	superior temporal gyrus	R		-4.51	63	-3	0
			**iRBD < PD^{RBD-}**						
LC_L	DMN	13	middle temporal gyrus	R		-4.11	51	-30	-6
	ECN	16	cerebellum_crus1	R		-3.97	15	-87	-30
	VIS	22	lingual gyrus	R		-4.64	6	-54	3
LC_R	SMN	18	superior temporal gyrus	L		-5.1	-51	-24	15

RSNs： resting-state networks; AAL: Anatomical Automatic Labeling; L/R: left/right; BA: Brodmann area; MNI: Montreal Neurological Institute; LC_L/R: left and right locus coeruleus; DMN: default mode network; ECN: executive control network; SAL: salience network; SMN: sensorimotor network; VIS:visual network； '<': the lower value of functional connectivity than other groups.

**Supplementary Table 4.** Decreased functional connectivity between pons and RSNs in PD patients.									
seeds	RSNs	cluster size	AAL	L/R	BA	peak intensity (T value)	MNI coordinate		
							X	Y	Z
**PD^{RBD-} < HC**									
pons	ECN	11	calcarine fissure and surrounding cortex	R		-4.46	18	-57	15
		12	inferior parietal, but supramarginal and angular gyri	R		-4.41	45	-45	45
		14	middle occipital gyrus	R		-4.37	36	-81	30
		11	superior frontal gyrus, dorsolateral	R		-3.77	24	9	45
		14	superior occipital gyrus	R		-4.06	30	-69	42
**PD^{RBD+} < HC**									
pons	DMN	18	cuneus	L		-4.34	-12	-66	24
		10	inferior frontal gyrus, triangular part	R		-4.13	51	18	6
		21/32	median cingulate and paracingulate gyri	R	31	-4.32	0	-30	45
		16	middle frontal gyrus	R		-5.36	39	3	39
		21	precentral gyrus	R		-5.06	36	0	39
		26	precuneus	R		-4.40	6	-21	42
		30	superior temporal gyrus	R		-4.21	54	-36	15
		22	supramarginal gyrus	R	40	-4.91	63	-39	24
	ECN	14	calcarine fissure and surrounding cortex	R		-3.97	18	-54	9
		32	inferior frontal gyrus, opercular part	R	9	-4.19	51	15	36
		19	inferior frontal gyrus, triangular gyrus part	R		-4.48	39	27	24
		38	inferior parietal, but supramarginal and angular gyri	R	40	-4.45	51	-48	48
		51	middle frontal gyrus	R		-4.74	27	9	54
		44	middle occipital gyrus	L	39	-4.62	-36	-81	21
		16	precentral gyrus	R		-4.70	42	3	27
		11/22	precuneus	R		-4.85	-12	-63	51
		34	superior frontal gyrus, dorsolateral	R		-4.83	21	12	45
	SAL	11	anterior cingulate and paracingulate gyri	R		-4.79	9	18	27
		22	inferior frontal gyrus, opercular part	R		-5.13	51	12	6
		36/35	median cingulate and paracingulate gyri	L/R	32	-5.04	3	15	42
		29	middle frontal gyrus	R		-4.64	30	33	24
		23	superior frontal gyrus, dorsolateral	R		-4.26	27	42	33
		19	superior frontal gyrus, dorsolateral_medial	L	24	-4.04	0	27	39
		16/12	supplementary motor area	L/R		-4.22	6	15	45
		44	supramarginal gyrus	R	40	-5.66	66	-33	33
	SMN	11	median cingulate and paracingulate gyri	L		-4.77	9	-9	42
		11	superior temporal gyrus	R	22	-4.68	54	12	3
		33/32	supplementary motor area	L/R	24/6	-4.24	12	-6	66
	VIS	25	middle occipital gyrus	R		-4.31	42	-78	18

RSNs： resting-state networks; AAL: Anatomical Automatic Labeling; L/R: left/right; BA: Brodmann area; MNI: Montreal Neurological Institute; LC_L/R: left and right locus coeruleus; DMN: default mode network; ECN: executive control network; SAL: salience network; SMN: sensorimotor network; VIS: visual network； ‘<’: the lower value of functional connectivity than other groups.
